# Periapical Lesions and Their Relationship to Schneider's Membrane in Cone-Beam Computed Tomography

**DOI:** 10.1155/2020/8450315

**Published:** 2020-03-09

**Authors:** César F. Cayo-Rojas, Leidy A. Begazo-Jiménez, Luighy B. Romero-Solórzano, Miriam K. Nicho-Valladares, Andrea Gaviria-Martínez, Luis A. Cervantes-Ganoza

**Affiliations:** ^1^Universidad Inca Garcilaso de la Vega, Investigation Institute, Lima, Peru; ^2^Universidad Inca Garcilaso de la Vega, Faculty of Stomatology, Lima, Peru; ^3^Universidad Nacional Federico Villarreal, Faculty of Dentistry, Lima, Peru

## Abstract

**Objective:**

To determine the relationship between the height of the periapical lesions adjacent to the maxillary sinus and the thickness of the Schneider membrane evaluated with cone-beam tomography. *Materials and Methods*. The universe was made up of 2432 tomography scans and a sample of 976, by systematic random sampling, and took into account those that presented any of the variables and/or both. For the relationship analysis, the sample was distributed according to sex, maxillary side, and age; it was formed between 18 and 86 years, in age groups of 18–36 years, 37–48 years, 49–59 years, and 60–86 years. The quantitative variables of the statistic descriptive analysis, hypothesis tests, and Spearman correlation were recorded.

**Results:**

A significantly low correlation (*p* < 0.010) was observed between the periapical lesions and the thickness of the Schneider membrane in women (rho = 0.38) and men (rho = 0.32); in the same way, a significantly low correlation was observed in the age groups of 18–36 years (rho = 0.27) and 37–48 years (rho = 0.28), while a significantly moderate correlation was observed in the age groups of 49–59 years (rho = 0.45) and 60–86 years (rho = 0.44), and with respect to the sides, a significantly low correlation (rho = 0.28) was obtained for the right side and a significantly moderate correlation (rho = 0.45) was obtained on the left side.

**Conclusion:**

We found that the height of the periapical lesions and the thickness of the Schneider membrane are significantly related according to age, sex, and maxillary side, this relationship being accentuated at an older age and on the left side.

## 1. Introduction

Schneider's membrane involvement may be due to different factors; however, in several studies, periapical lesions have been identified as the main cause of this [1–6]. This can be observed by means of auxiliary examinations, such as cone-beam tomography, in such a way that if we find periapical lesions (PL) adjacent to the maxillary sinus; in many cases, they produce some alteration directly to Schneider membrane (SM), and this relation can even be observed clinically in the patient as is the case of sinusitis as a result of an infectious complication of the periapex. The most frequent diseases affecting the maxillary sinus are acute rhinosinusitis (ARS), chronic rhinosinusitis (CRS), pseudocyst, retention cyst, and mucocele. The persistence of the chronic inflammation can promote an epithelial damage with metaplastic changes, epithelial shedding, loss of ciliated cells, and increase of goblet cells, leading to an impaired function [7].

However, in everyday clinical practice, mucosal thickening of the maxillary sinus is a common radiographic finding in asymptomatic patients; therefore, mucosal lining of more than 4 mm is considered to be pathological [8]. Computed tomography (CT) is regarded as the gold standard for the diagnosis of problems related to sinuses because it provides multiple sections through the sinus at different planes and allows both bones and soft tissue to be seen. Recently, cone-beam CT has been introduced for dental and maxillofacial imaging, and it is reliable for the evaluation of structures within the region, including the maxillary sinus [9–11]. Thus, cone-beam tomographies are currently used by many dentists because it allows observing these pathological processes in three dimensions with a minimum distortion percentage (up to 0.01%), as opposed to orthopantomographies that offer a distortion of 20%. Another advantage of using tomographies is that we can make exact measurements of the findings and make statistical analysis without risk of obtaining biases. This is why this research aims to evaluate whether or not there is a correlation between Schneider membrane and adjacent periapical lesions using cone-beam tomography as an instrument because SM is richly vascularized at the level of its own lamina, presenting cells of the connective tissue that could react with inflammatory chemical mediators that in turn stimulate plasma extravasation of the blood vessels below the epithelium, thus causing a thickening of SM.

Among the studies that found a significant correlation between the thickening of the Schneider membrane and the periapical lesions, we have that of Aksoy and Orhan [1] because they demonstrated a direct relationship between both variables associated with age, sex, and teeth missing; Nunes et al. [3] showed in their study that the posterior teeth with periapical lesions had the highest frequency of sinus anomalies, Sheikhi et al. [5] in their study showed that Schneider's membrane thickening was directly related with periapical bone loss, and finally Shanbag et al. [6] in their study concluded that the thickening of the Schneider membrane is significantly related to periapical lesions more frequently in the male sex.

On the other hand, some studies such as the one carried out by Nascimento et al. [2] found that there was no statistically significant relationship between the thickening of the Schneider membrane and some periapical pathologies. Likewise, Bloque and Dastoury [4] in their study found that healthy teeth and teeth with some pathology did not show significant differences regarding Schneider membrane thickening.

Because of these results, the decision was made to expand the sample, as suggested by numerous studies. The main objective was to determine the relationship between the height of the periapical lesions adjacent to the maxillary sinus and the thickness of the Schneider membrane evaluated with cone-beam tomography and secondary objective to establish this relationship according to sex, maxillary side, and age group of 18–36 years, 37–48 years, 49–59 years, and 60–86 years.

## 2. Materials and Methods

This study was cross-sectional, retrospective, and correlational.

The universe consisted of 2432 cone-beam tomographies of a population originally from Lima, Peru and a sample of 976; for the calculation of this one, a pilot study was made with 30 samples, and a standard deviation of 1.91 was obtained, and an error of 0.12 mm was considered [12]. For the relationship analysis, the sample was distributed according to sex, maxillary side, and age; it was formed between 18 and 86 years, in age groups of 18–36 years, 37–48 years, 49–59 years, and 60–86 years.

The experiment and data recording were performed at the Life 3D Imaging Center™ and the Maxillofacial Diagnostic Institute (MDI)™.

The variables used in the study were as follows:

  Periapical lesion (PL), whose indicator to evaluate was the height
  Schneider membrane (SM), whose indicator to evaluate was width (mm)

### 2.1. Technique and Procedures for Obtaining Information

The method of sampling was systematic randomization.

The length of the respective variables SM and PL was measured by the investigator after calibration (intraclass correlation coefficient), interexaminer (0.92 and 0.94), and intraexaminer (0.95 and 0.95, respectively) with a specialist in oral maxillofacial radiology with more than 20 years of experience.

Data records from the Life 3D Imaging Center™ and the Maxillofacial Diagnostic Institute (MDI)™ were accessed to collect and identify those scans that presented one or both variables, and the data were entered into an *ad hoc* file. The information was extracted from the cone-beam tomography database taken with the PaX-i 3D Smart® equipment from Vatech (Korea) and Promax 3D® from Planmeca (Finland), from the Life Image Center 3D™ and the Maxillofacial Diagnostic Institute (IDM)™, respectively. MS and LP measurements of adjacent parts were taken using Ez 3D plus® software version 1.0.9 and Romexis® version 5.0.0. The parameters used in the cone-beam tomographs collected were 90 Kv and 8 mA. With a 360-degree rotation and scan exposure time of 10 to 20 seconds, the FOV (field of view) was 100 *∗* 100 mm and 40 *∗* 50 mm; sagittal cuts were made to these tomographic volumes in order to measure the thickness of the Schneider membrane and the height of the periapical lesions.

The data were stored in Microsoft Excel 2016 software and imported with *SPSS* (*statistical package for the social sciences*) version 24 using a classification table with descriptive values of central tendency and dispersion. The nonparametric Spearman correlation test was used to contrast the correlation hypothesis between study variables. This test was used because the data obtained did not present a normal distribution when doing the Kolmogorov–Smirnov test (*p* > 0.05). To compare the measurements (mm) of the periapical lesion and the Schneider membrane, according to sex, age, and side, nonparametric Mann–Whitney *U* tests were used for two independent samples, Kruskall–Wallis test for more than two independent samples, and postestimation of Bonferroni for multiple comparisons.

All statistical evidence were contrasted at a 95% confidence level and 5% significance level.

As an ethical consideration, the data were kept in absolute confidence and no names were registered in such a way that there is no risk of violating patient confidentiality. The information was only used for the purpose of the present investigation.

## 3. Results

Table 1 shows descriptive values for the height of periapical lesions, where values of 1.72 mm ± 1.99 mm with a median of 1.40 mm are observed for those of the female sex and 2.12 mm ± 2.06 mm with a median of 2.00 mm for the male sex. In addition, those of the male sex present a height of periapical lesions significantly greater than those of the female sex.

With respect to the age groups, the 18–36-year group presents average values for the height of periapical lesions of 1.52 mm ± 1.84 mm with a median of 1.30 mm, the 37–48-year group of 2.03 mm ± 1.85 mm with a median of 2.0 mm, the 49–59-year group of 2.06 mm ± 2.10 mm with a median of 1.80 mm, and the 60–86-year group of 1.93 mm ± 2.29 mm with a median of 1.50 mm. In addition, there are significant differences between at least two of the age groups.

On the right side, we found mean values of the height of the periapical lesions of 1.89 mm ± 2.05 mm with a median of 1.70 mm, and for the left side, 1.89 mm ± 2.03 mm with a median of 1.80 mm.

In Table 2, it can be seen that the age group of 37 to 48 years and that of 49 to 59 years presented a significantly higher periapical lesion height (*p*=0.002 and *p*=0.008, respectively), compared with the age group from 18 to 36 years old.

Table 3 shows descriptive values of Schneider's membrane thickness, where the female sex is observed with values of 2.60 mm ± 2.93 mm with 2.00 mm median and the male sex with values of 3.59 mm ± 3.36 mm with a median of 3.10 mm. In addition, it is observed that the male sex has significantly greater Schneider's membrane thickness, compared with the female sex (*p* < 0.05).

With respect to the age groups, the 18–36-year group presents average values for Schneider's membrane thickness of 3.10 mm ± 3.37 mm with a median of 2.75 mm, the 37–48-year group of 2.97 mm ± 2.94 mm with a median of 2.70 mm, the 49–59-year group of 2.75 mm ± 2.66 mm with a median of 2.50 mm, and the 60–86-year group of 3.27 mm ± 3.61 mm with a median of 2.00 mm.

On the right side, we found mean values of Schneider's membrane thickness of 3.18 mm ± 3.21 mm with a median of 2.70 mm, and for the left side, 2.81 mm ± 3.07 mm with a median of 2.20 mm.

Table 4 shows a significant low degree of correlation (*p* < 0.010) between periapical lesions and Schneider membrane thickness for females (rho = 0.38) and males (rho = 0.32).

Table 5 shows a significant degree of correlation (*p* < 0.010) between periapical lesions and Schneider's membrane thickness, the 18–36-year group with low level of correlation (rho = 0.27); the 37–48-year group with low level (rho = 0.28); the 49–59-year group with moderate level (0.45); and the 60–86-year group with moderate level (rho = 0.44).

Table 6 shows a significant degree of correlation (*p* < 0.010) between periapical lesions and Schneider's membrane thickness, for the right side with low level of correlation (rho = 0.28) and the left side with moderate level (rho = 0.45).

## 4. Discussion

Aksoy and Orhan [1] evaluated tomography scans of 294 patients and determined that the thickening of the sinus mucosa was greater than 2 mm with periapical lesions coinciding with the values obtained in this study because values greater than 3 mm were obtained for both sexes; on the other hand, Aksoy and Orhan [1] obtained a significant correlation between SM and PL thickening according to age and sex, differing with this study because it was determined that if there is a correlation between LP and MS, but this is not significant for the sex variable, however, it coincides as to the moderate correlation for age for the groups of 48–59 and 60–86 years.

Shanbhag et al. [6] in a sample of 243 patients showed significant associations between SM thickening and PL with respect to male sex, age >60 years, being similar to the results of this research in terms of correlation with the age variable because in both sexes, patients over 60 years had a moderate correlation, however, differs in terms of correlation with the sex variable where low association was obtained.

Sheikhi et al. [5] and Nunes et al. [3] corroborate the aforementioned investigations, and all of them agreed that if there is an association between PL and SM thickening, the present study differs from the previous ones in terms of the degree of correlation between PL and SM thickness as it was obtained that there is indeed a correlation but it was low, it should be noted that the previous studies had a much smaller sample than the present study.

In the study carried out by Nascimiento et al. [2] in 400 patients, it was observed that SM thickening is associated with PL higher in men, while in this study with a sample of 976, it is corroborated that there is association between SM and PL, but to a low degree for both sexes equally.

With respect to the study of Bloque and Dastoury [4] with a sample of 1662, they obtained that SM thickening can give almost similar results in teeth with PL and without PL; however, the conclusions of these authors did not obtain the product of a correlation analysis of Spearman. In this study, when making the correlation analysis, it was obtained that there is significant association between SM thickening and PL, but of low degree for both sexes, moderate for ages 49 and up, and in the same manner for the location on the left side.

This study demonstrates that the male sex presents a greater Schneider's membrane thickness with respect to the female sex, corroborating the results obtained by Kalyvas et al. [7] and Yildirim et al. [13].

This association of SM and PL thickening may be due to the fact that the maxillary sinus is made up of a pseudostratified epithelium with hair cells adjacent to its own richly vascularized lamina that has contact with the bone tissue that borders the apexes of the posterior teeth, existing dentosinusal proximity because the floor of the maxillary sinus is the declivity zone, which at the level of the first and second molars is more pronounced. In this way, as there is greater height (which would bring it closer to the maxillary sinus) of the periapical lesions in the area, the natural response to this injury would cause chemical mediators to be released from the inflammation by the cells of the connective tissue present in the lamina itself of the SM and also from the white blood cells present in the medullary areas of the spongy bone present between the PL and the SM [10, 14, 15].

In conclusion, the PL height values are significantly higher in the male sex; on the other hand, people aged 37 to 59 years had a significantly higher PL height compared with the younger ones, and with respect to the side, no differences were demonstrated. Regarding SM, the male sex presented a significantly greater thickness, without finding differences between the age groups and both sides. It can also be said that there is a low significant correlation between the height PL and the thickness SM for both sexes, and in the same way, there is a low correlation between the height PL and the thickness SM for the age groups of 18 to 48 years and moderate level for age groups 49–86 years. With respect to the side, there is a low correlation between the height PL and the thickness SM for the right side and moderate for the left side.

Based on the results obtained, it is recommended to carry out studies on the relationship between the SM thickening and the PL height according to the type of tooth using cone-beam tomography.

## Figures and Tables

**Table 1:** Descriptive values of the height of periapical lesions (mm) adjacent to the maxillary sinus according to sex, age group, and side.

Parameter	Categories	*n*	Half	95% CI		Median	SD	*p* value
				LL	UL			
Sex	Female	567	1,72	1,56	1,89	1,40	1,99	<0.05^*∗*^^a^
	Male	409	2,12	1,92	2,32	2,00	2,06	
	Total	976	1,89	1,76	2,02	1,80	2,03	

Age group	18–36	246	1,52	1,29	1,75	1,30	1,84	0.001^*∗*^^b^
	37–48	251	2,03	1,80	2,26	2,00	1,85	
	49–59	253	2,06	1,80	2,32	1,80	2,10	
	60–86	226	1,93	1,63	2,23	1,50	2,29	
	Total	976	1,89	1,76	2,02	1,80	2,03	

Side	Right	525	1,89	1,71	2,06	1,70	2,05	0.945
	Left	451	1,89	1,70	2,07	1,80	2,01	
	Total	976	1,89	1,76	2,02	1,80	2,03	

CI, 95% confidence interval; LL, lower limit; UL, upper limit; SD, standard deviation; based on the ^a^Mann–Whitney *U* test; ^b^Kruskall–Wallis test; ^*∗*^significant differences (*p* < 0.05).

**Table 2:** Multiple height comparisons of periapical lesions between age groups.

Age group	37–48	49–59	60–86
18–36	*p*=0.002^*∗*^	*p*=0.002^*∗*^	*p*=0.546
37–48	—	*p*=1.000	*p*=1.403
49–59	—	—	*p*=0.896
60–86	—	—	—

Based on the Bonferroni test, ^*∗*^significant difference (*p* < 0.05).

**Table 3:** Descriptive values of Schneider's membrane thickness (mm) by sex, age group, and side.

Parameter	Categories	*n*	Half	95% CI		Median	SD	*p* value
				LL	UL			
Sex	Female	567	2,60	2,36	2,84	2,00	2,93	<0.05^*∗*^^a^
	Male	409	3,59	3,26	3,92	3,10	3,36	
	Total	976	3,01	2,82	3,21	2,50	3,15	

Age group	18–36	246	3,10	2,68	3,52	2,75	3,37	0.935^b^
	37–48	251	2,97	2,60	3,33	2,70	2,94	
	49–59	253	2,75	2,42	3,08	2,50	2,66	
	60–86	226	3,27	2,79	3,74	2,00	3,61	
	Total	976	3,01	2,82	3,21	2,50	3,15	

Side	Right	525	3,18	2,91	3,46	2,70	3,21	0.053
	Left	451	2,81	2,53	3,10	2,20	3,07	
	Total	976	3,01	2,82	3,21	2,50	3,15	

CI, 95% confidence interval; LL, lower limit; UL, upper limit; SD, standard deviation; based on the ^a^Mann–Whitney *U* test; ^b^Kruskall–Wallis test; ^*∗*^significant differences (*p* < 0.05).

**Table 4:** Correlation between the length and the height of the periapical lesions adjacent to the maxillary sinus and the thickness of Schneider's membrane, according to sex.

Sex	Parameter	*n*	Half	Median	Rho	*p* value
Female	HPL	567	1.72	1.40	0.38^*∗∗*^	0.001
	SMT	567	2.60	2.00		

Male	HPL	409	2.12	2.00	0.32^*∗∗*^	0.001
	SMT	409	3.59	3.10		

HPL, height of periapical lesions; SMT, Schneider membrane thickness; Rho, Spearman correlation coefficient; *p* value <0.010.

**Table 5:** Correlation between the length and the height of the periapical lesions adjacent to the maxillary sinus and the thickness of the Schneider membrane, according to the age group.

Age group	Parameter	*n*	Half	Median	Rho	*p* value
18–36	HPL	246	1.52	1.30	0.27^*∗∗*^	0.001
	SMT	246	3.10	2.75		

37–48	HPL	251	2.03	2.00	0.28^*∗∗*^	0.001
	SMT	251	2.97	2.70		

49–59	HPL	253	2.06	1.80	0.45^*∗∗*^	0.001
	SMT	253	2.75	2.50		

60–86	HPL	226	1.93	1.50	0.44^*∗∗*^	0.001
	SMT	226	3.27	2.00		

HPL, height of periapical lesions; SMT, Schneider membrane thickness; Rho, Spearman correlation coefficient; *p* value <0.010.

**Table 6:** Correlation between the height of the periapical lesions adjacent to the maxillary sinus and the thickness of the Schneider membrane, according to side.

Side	Parameter	*n*	Half	Median	Rho	*p* value
Right	HPL	525	1.89	1.70	0.28^*∗∗*^	0.001
	SMT	525	3.18	2.70		

Left	HPL	451	1.89	1.80	0.45^*∗∗*^	0.001
	SMT	451	2.81	2.20		

HPL, height of periapical lesions; SMT, Schneider membrane thickness; Rho, Spearman correlation coefficient; *p* value <0.010.

## Data Availability

The data used are in complementary files of this article.
